# Biological and clinical significance of epigenetic silencing of *MARVELD1* gene in lung cancer

**DOI:** 10.1038/srep07545

**Published:** 2014-12-18

**Authors:** Ming Shi, Shan Wang, Yuanfei Yao, Yiqun Li, Hao Zhang, Fang Han, Huan Nie, Jie Su, Zeyu Wang, Lei Yue, Jingyan Cao, Yu Li

**Affiliations:** 1 School of Life Science and Technology, Harbin Institute of Technology, Harbin, China; 2 Department of Medical Oncology, Harbin Medical University Cancer Hospital, Harbin, China

## Abstract

Epigenetic silence in cancer frequently altered signal-transduction pathways during the early stages of tumor development. Recent progress in the field of cancer epigenetics has led to new opportunities for diagnosis and treatment of cancer. We previously demonstrated that novel identified nuclear factor MARVELD1 was widely expressed in human tissues, but down-regulated by promoter methylation in multiple cancers. This study was carried out to determine the biological and clinical significance of *MARVELD1* gene silencing in lung cancer. Here, we found the reduced MARVELD1 expression significantly correlated with diagnostic histopathology and malignant degree of lung cancers. DNA hypermethylation and histone deacetylation synergistically inactivated *MARVELD1* gene in lung cancer cells. Moreover, MARVELD1 modulated the efficiency of nonsense-mediated mRNA decay (NMD) through interaction with NMD core factor SMG1. The decreased MARVELD1 level in lung cancer reduces NMD efficiency through diminishing the association between NMD complex component UPF1/SMG1 and premature termination codons containing mRNA (PTC-mRNA). The results suggested that MARVELD1 silencing is an appealing diagnostic biomarker for lung cancer and epigenetic silencing of *MARVELD1* gene links with the regulatory mechanism of NMD pathway in lung cancer, which may be required for tumorigenesis.

Lung Cancer is the leading cause of cancer-related death. Traditional treatments are not effective in lung cancer, and 5-year survival rate of lung cancer patients is lower than many other cancers. The related mechanism has become obstacles for the success of lung cancer therapy. Tumor suppressor genes usually protect normal cells from progressing to cancer^1^. However, these genes in cancer cells often suffer genetic mutations and aberrant epigenetic modifications^2^,^3^. The mutations generate mRNA harboring premature termination codons (PTCs) that are targets of nonsense-mediated mRNA decay (NMD) pathway^4^. Hence NMD regulation and aberrant epigenetic modifications contribute to oncogenesis^5^,^6^,^7^.

NMD is an mRNA surveillance pathway that eliminates aberrant mRNA transcript containing PTCs, and prevents the synthesis of potentially toxic truncated proteins^8^,^9^. In mammalian cells, the main NMD targets are aberrant PTC-containing transcripts owing to error-prone transcription and mistakes during pre-mRNA processing^10^,^11^,^12^. In addition to mRNA quality control, NMD also couples with alternative splicing to regulate the abundance of endogenous mRNA in specific cellular event^13^. A central component of NMD pathway is the evolutionarily conserved DNA/RNA helicase UPF1^14^. Another core kinase of the NMD machinery is SMG1 that phosphorylates the conserved UPF1 effector to activate NMD^15^. Biochemical evidences imply that both UPF1 and SMG1 shuttle between cytoplasm and nucleus^16^,^17^. The biological roles and core factors of NMD pathway have been reported; however, the regulatory process of NMD in lung cancer has not been characterized.

MARVELD1 (MARVEL domain containing 1) is a novel identified nuclear factor. We previously showed that MARVELD1 is widely expressed in normal human tissues and is down-regulated in primary breast carcinomas^18^. Furthermore, MARVELD1 has recently been implicated in growth inhibitory effects of tumor cell and regulation of cell adhension^19^,^20^. In the present study, we tested the expression pattern of MARVELD1 and the epigenetic status of *MARVELD1* promoter in lung cancer. We found the decreased expression of MARVELD1 was due to DNA hypermethylation and histone deacetylation of *MARVELD1* promoter. Remarkably, silencing of MARVELD1 expression by aberrant epigenetic modifications reduced the efficiency of NMD and represented a potential biomarker in lung cancer.

## Results

### Low expression of MARVELD1 in lung cancer tissues and cell lines

**Figure 1:**
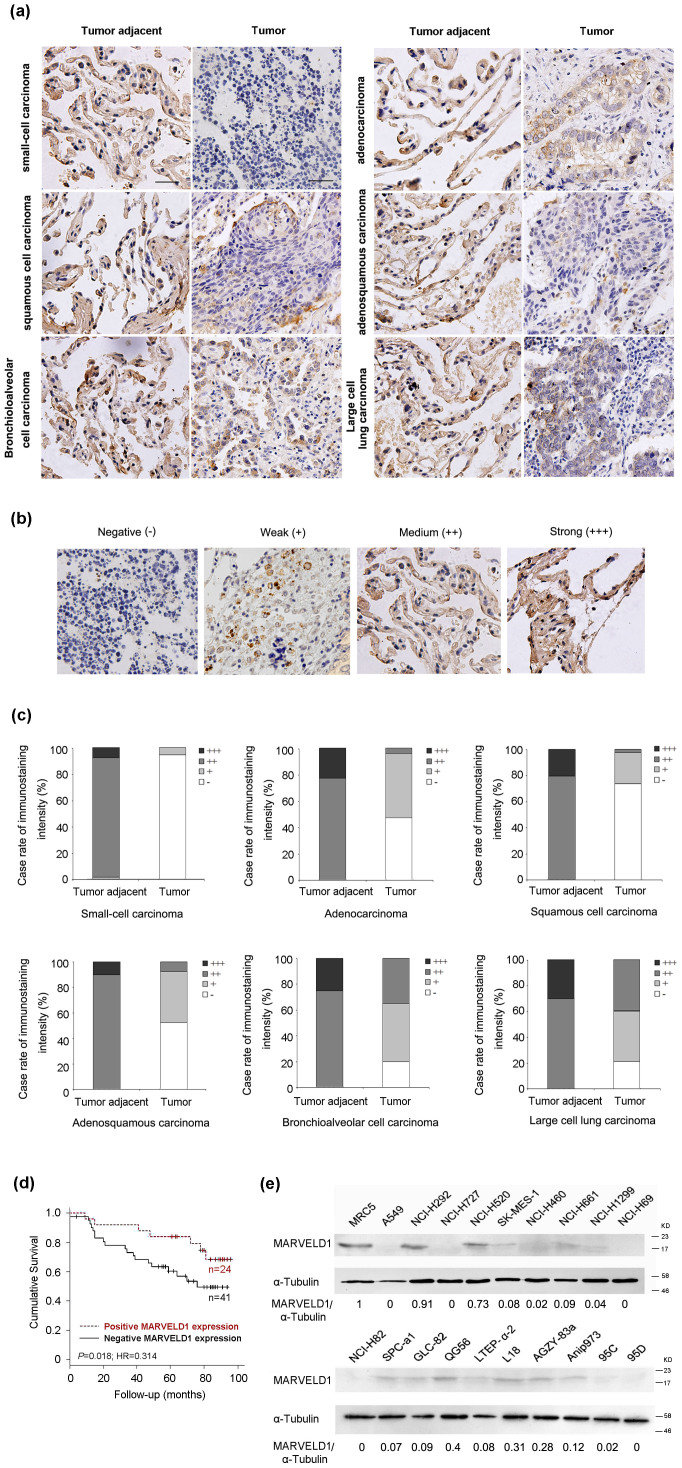
MARVELD1 expression in lung cancer tissues and cell lines. (a) Immunohistochemical analysis showed MARVELD1 level in lung cancer tissues and adjacent lung tissues. (b), (c) Classification of samples according to the intensity of staining of MARVELD1 expression. (d) Kaplan-Meier survival plot showing the cumulative survival of lung cancer patients who displayed negative MARVELD1 expression in tumor tissues and patients with positive MARVELD1 expression in tumor tissues (*P* = 0.018, HR = 0.314, 95% CI, 0.12–0.82). (e) Western blotting analysis showed MARVELD1 level in various lung cancer cell lines.

To investigate the clinical significance of MARVELD1 in lung cancer, immunohistochemical analysis was performed in total 194 cases of lung cancer specimen, including 55 cases of small cell lung cancer (SCLC) specimen, 35 cases of adenocarcinoma specimen, 44 cases of squamous cell carcinoma specimen, 20 cases of adenosquamous carcinoma specimen, 20 cases of bronchioalveolar cell carcinoma specimen, 20 cases of large cell lung carcinoma specimen and their adjacent lung tissues with MARVELD1 specific antibody. As shown in Figure 1a, we found that MARVELD1 was highly expressed in adjacent noncancerous lung tissues compared with tumor tissues. Based upon the staining intensity, we classified the samples into four groups with increasing intensity of staining from negative (−) to strong (+++) (Figure 1b). MARVELD1 expression in all the tested tumor adjacent control cases were positive (++ or +++); However, its expression in small cell lung cancer tissues was negative (94.5%; 52/55) or very weak (5.5%; 3/55). MARVELD1 expression was also dramatically reduced, either negative (−) or weak (+) expression, 91.4% in adenocarcinoma tissues, 97.7% in squamous cell carcinoma, 90% in adenosquamous carcinoma, 65% in bronchioalveolar cell carcinoma, and 60% in large cell lung carcinoma (Figure 1c). The reduced expression of MARVELD1 was confirmed in lung cancer data from The Cancer Genome Atlas (TCGA) dataset. Analysis of tumor/nontumor adjacent tissue (T/N) ratios for MARVELD1 expression in 25 NSCLC patients revealed that MARVELD1 expression was decreased in 72% lung cancer tissues (Figure S1a).

Furthermore, in NSCLC, MARVELD1 expression level seems to be related to the grade of the disease development. We found 63.6% (21 out of 33 NSCLC) in grade I tumor showed the reduced MARVELD1 expression, but 96.9% (62 out of 64) in the advanced grades (grade II to III) (Table S2, *P* < 0.001). Kaplan-Meier survival analysis showed that negative MARVELD1 expression correlated with reduced patient survival compared with positive MARVELD1 expression in tumor tissues (*P* = 0.018; Hazard Ratio (HR) = 0.314; 95% Confidence Interval (CI), 0.12–0.82) (Figure 1d). However, no correlation between MARVELD1 expression and other clinical parameters (e.g., age and gender) was observed.

This phenomenon was further confirmed in lung cancer cell lines. The human lung fibroblast cell line MRC-5 and 19 lung carcinoma cell lines were tested by western blotting analysis. Shown as Figure 1e, MARVELD1 level was significantly down-regulated in lung cancer cell line A549, NCI-H727, SK-MES-1, NCI-H460, NCI-H1299, NCI-H69, NCI-H82, LTEP-α-2, 95C, 95D, NCI-H661, SPC-a1, GLC-82, QG56, L18, AGZY-83a and Anip973, slightly down-regulated in NCI-H520, and not altered significantly in NCI-H292 compared to MRC-5 cells. Negative expression of MARVELD1 was observed in all 2 SCLC cell lines NCI-H69 and NCI-H82 (100%), but in 2 (A549 and NCI-H727) out of 17 NSCLC cell lines (11.8%). Compared with human lung fibroblast cell line MRC-5, 15 out of 17 NSCLC cell lines (88.2%) showed significantly reduced MARVELD1 expression.

Taken together, these findings suggested that silencing expression of *MARVELD1* gene may be required for lung tumorigenesis and maintenance of malignant phenotypes. Thus, silencing expression of MARVELD1 has a potential to be developed as a biomarker for malignant phenotype of lung cancer.

### Hypermethylation of *MARVELD1* promoter in lung cancer cells

**Figure 2:**
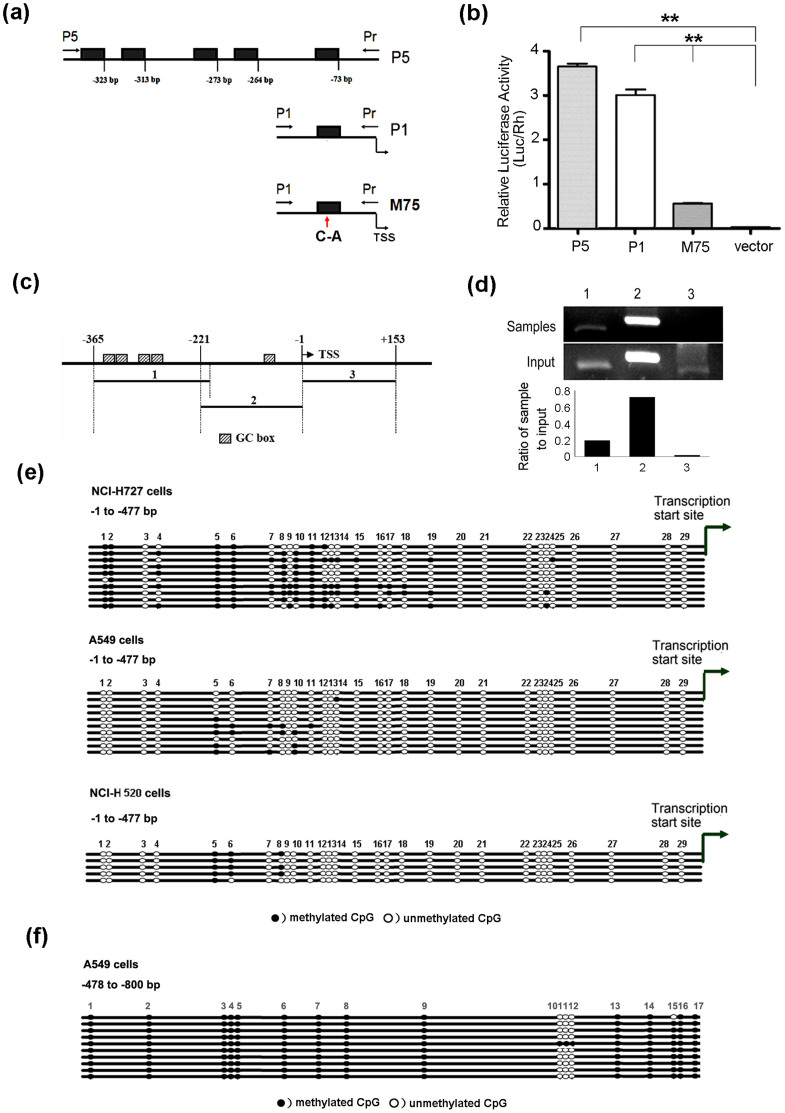
Promoter activity and methylation analysis of *MARVELD1* gene in lung cancer cell lines. (a) Model of truncated *MARVELD1* promoter and site-directed mutation on *MARVELD1* promoter. (b) The effect of truncation and site-directed mutation on *MARVELD1* promoter activity. The truncated and mutated promoter fragments were introduced into the firefly luciferase reporter plasmid pGL3-basic (Luc). The luciferase activity was assayed 24 h post-transfection. Values are means ± S.D. obtained from three independent experiments. (c) Structure mode of *MARVELD1* promoter for ChIP analysis. (d) Chromatin immunoprecipitation (IP) assay using H520 cell lysates. Chromatin was immunoprecipitated using antibody against Sp1. Full length gel was shown in supplementary figure 6A. (e) Bisulfite sequencing PCR and DNA sequence analysis showed methylation status of MARVELD1 proximal promoter (−1 to −477 bp) in lung cancer cell line NCI-H727, A549 and NCI-H520. (f) Bisulfite sequencing PCR and DNA sequence analysis showed methylation status of MARVELD1 distal promoter region (−478 to −800 bp) in lung cancer cell line A549.

To understand the mechanisms involved in regulation of MARVELD1 expression in lung cancer, we analyzed transcriptional regulation of *MARVELD1* gene. Bioinformatics analysis showed that MARVELD1 promoter was devoid of distinct TATA box and CAAT box, while there were five GC boxes in the promoter region (−1 to −477 bp) of *MARVELD1* gene. The promoter activity of GC boxes in MARVELD1 promoter was examined by luciferase assay. As shown in Figure 2a and 2b, P5 promoter containing 5 GC boxes exhibited the highest promoter activity, and P1 promoter containing only 1 GC box also displayed high activity, which indicated the GC box in P1 promoter played a key role in MARVELD1 promoter. To confirm the role of GC box in P1, M75 promoter with a site-directed mutation (C-75A) was constructed (Figure S1b). The activity of promoter was abolished by the site-directed mutation of GC box in M75.

Since GC box-dependent promoter specifically binds to transcription factor Sp1, we next investigated the interaction of Sp1 with GC box by Chromatin immunoprecipitation (ChIP) assay. *MARVELD1* promoter was divided into 2 parts (4 GC boxes in the 1st part −365 ~ −200 bp, 1 GC box in the 2nd part −221 ~ −1 bp). Control sequence was from MARVELD1 encoding region +1 ~ +153 bp (Figure 2c). DNA fragmentation was generated from genome DNA (Figure S2). Shown as Figure 2d, ChIP assay demonstrated that the 2nd part of *MARVELD1* promoter (P1 promoter) interacted with transcription factor Sp1. These results indicated that Sp1 binding site mainly located at the position of −75 GC box, and MARVELD1 promoter was dependent on Sp1 for its activity.

In vitro, we further investigated the methylation status of *MARVELD1* promoter in lung cancer cell lines by bisulfite sequencing PCR and DNA sequence analysis. The hypermethylation of *MARVELD1* promoter was discovered and the methylation pattern in low MARVELD1-expressing NCI-H727 and A549 cells are shown in Figure 2e and 2f. Twenty nine CpG sites within 477 bp promoter region of MARVELD1 in NCI-H727 were analyzed. It was highly methylated at the CpG sites of *MARVELD1* promoter in NCI-H727, and 69% GC sites (20/29) were methylated. Specifically, the number of CpG sites with highly (≥80%), intermediately (20–80%), and lowly (≤20%) methylated levels was 7 (24.1%), 8 (27.6%), 14 (48.3%), respectively (Figure 2e). The methylation pattern of *MARVELD1* promoter in A549 cells was quite different from NCI-H727 cells. There were rare methylated CpG sites in MARVELD1 proximal promoter (−1 to −477 bp) of A549 cells, while extremely high methylation level in the distal promoter region (−478 to −800 bp) (Figure 2f). Whereas in relatively high MARVELD1-expressing NCI-H520 cells, the *MARVELD1* promoter was lowly methylated in promoter region (Figure 2e).

### Effect of 5-aza-CdR and TSA on MARVELD1 expression in lung cancer cells

**Figure 3:**
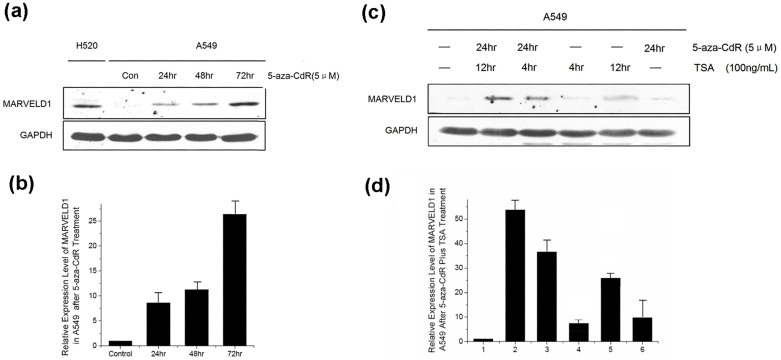
Effect of epigenetic inhibitors on expression of *MARVELD1* gene in lung cancer cells. (a), (c) Western blotting analysis showed the effect of 5-aza-CdR (a) and the effect of 5-aza-CdR and TSA (c) on MARVELD1 expression in A549 cells. Full length blots were shown in supplementary figure 6b, 6c. (b), (d) Relative intensities of bands in western blotting were determined by using NIH image software.

DNA methyltransferase inhibitor 5-Aza-2′-deoxycytidine (5-aza-CdR) can reverse the abnormal CpG islands hypermethylation and recover the expression of the silenced tumor suppressor genes. Therefore, we investigated the influence of 5-aza-CdR on the expression of *MARVELD1* gene in lung cancer cells. MARVELD1 expression was obviously enhanced by 5 μM of 5-aza-CdR in A549 cells in a time-dependent manner. H520 with high MARVELD1 expression acted as a positive control (Figure 3a). MARVELD1 level in 5-aza-CdR treated A549 cells was significantly increased during 24 to 72 h of treatment as compared with untreated group. 72 h of 5-aza-CdR treatment induced more MARVELD1 expression than shorter incubation period (24 and 48 h) (Figure 3b). We also detected MARVELD1 expression in different concentration of 5-aza-CdR, and found an apparent dose-dependent increase of MARVELD1 level (Figure S3).

Besides DNA methylation, histone acetylation also plays a vital role in the regulation of some genes expression. To analyze the synergistic effects of epigenetic inhibitors on MARVELD1 gene expression, western blotting was performed using A549 cells treated with 5-aza-CdR and specific inhibitor of histone deacetylase trichostatin A (TSA). Shown as Figure 3c, MARVELD1 expression was up-regulated after the cells treated with 5-aza-CdR, TSA, alone or combination. The effect of TSA on MARVELD1 expression was similar as 5-aza-CdR treatment. The combination of 5-aza-CdR and TSA appeared to produce a synergistic activation of *MARVELD1* gene (Figure 3d). These results indicated that DNA demethylation worked in harmony with histone acetylation to control the expression of MARVELD1 in lung cancer cells.

### MARVELD1 interacts with NMD factor SMG1

**Figure 4:**
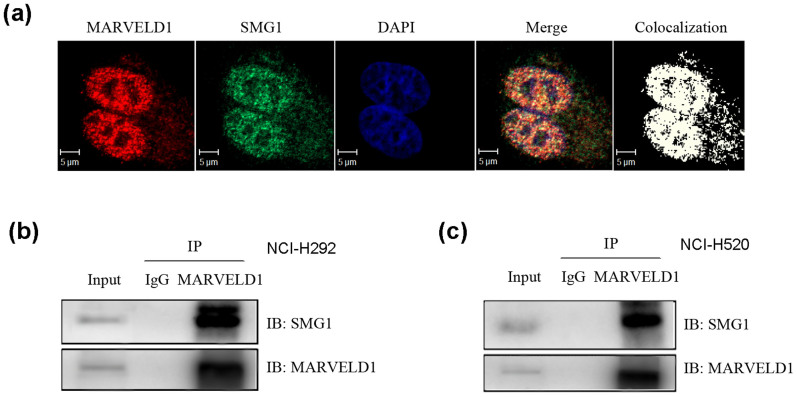
Identification of the interaction between MARVELD1 and SMG1. (a) Immunofluorescence staining analysis showed the colocalization of MARVELD1 and SMG1. (b), (c) Co-IP analysis showed the interaction between MARVELD1 and SMG1 in NCI-H292 (b) and NCI-H520 (c) cells. MARVELD1 and SMG1 were immunoprecipitated using antibody against MARVELD1. Anti-IgG was used as a negative control. Full length blot was shown in supplementary figure 6d.

Previous results showed that MARVELD1 associated with Importin β1, which has been suggested to involve in NMD regulation^21^,^22^. To test the role of MARVELD1 in NMD pathway, we detected the localization of MARVELD1 and NMD factor SMG1 in MARVELD1-overexpressed A549 cell line by immunofluorescence staining. As shown in Figure 4a, MARVELD1 colocalized with SMG1 completely. We further detected the potential interaction between endogenous MARVELD1 and SMG1 in lung cancer cell line NCI-H292 by using Co-IP followed by western blotting analysis. NCI-H292 whole cell lysates were prepared and immunoprcipitation was performed with a control rabbit antibody (IgG) and MARVELD1 antibody. Shown as Figure 4b, MARVELD1 and SMG1 were seen in the cell lysate of NCI-H292, and both proteins were immunoprecipitated with MARVELD1 antibody from cell lysate. The interaction was also confirmed in lung cancer cell line NCI-H520 (Figure 4c). Thus, these results indicated that MARVELD1 might be involved in NMD regulation through association with SMG1.

### MARVELD1 is required for efficient NMD

**Figure 5:**
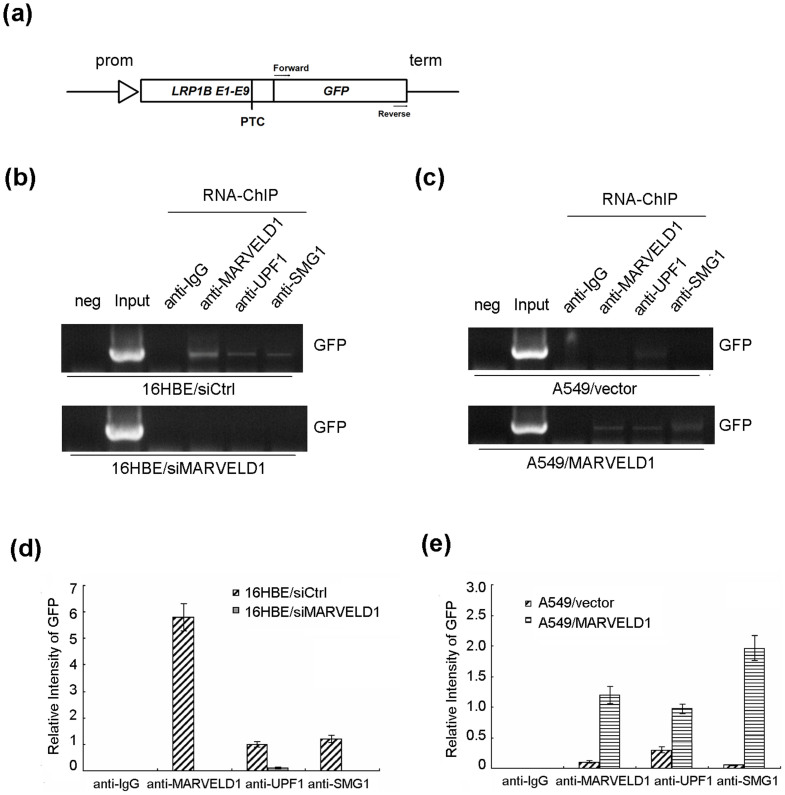
MARVELD1 functions in NMD pathway. (a) Diagram of NMD reporter plasmid with a PTC in *LRP1B* gene exons 1–9 from lung cancer cell QG56 and GFP-coding region. (b), (c) The association of GFP-tagged PTC-mRNA and proteins MARVELD1, UPF1 or SMG1 in 16HBE and A549 cells was evaluated in RNA-ChIP assay by using MARVELD1, UPF1 or SMG1 specific antibody. H2O (neg) acted as a negative control in PCR assay. One percent of supernant (Input) acted as a positive control. Anti-IgG acted as a negative control in RNA-ChIP assay. Immunoprecipitated RNA was subjected to RT-PCR using primers specific to GFP. Full length gels were shown in supplementary figure 6e. (d), (e) Statistical analysis showed the amount of PTC-mRNA immunoprecipitated with IgG, MARVELD1, UPF1 and SMG1 specific antibodies.

To examine whether MARVELD1 affected NMD, we generated a NMD reporter plasmid carrying a PTC derived from deletion mutation of endogenous *LRP1B* gene exons 1–9 in lung cancer cell QG56 (Figure S4); GFP-coding region was inserted at C-terminal of *LRP1B* gene (Figure 5a). The plasmid was transfected into immortal human bronchial epithelial cells 16HBE that expressed high level of MARVELD1 and human lung adenocarcinoma epithelial cells A549. MARVELD1 level in these cells was analyzed by western blotting (Figure S5). Two days post-transfection, we carried out RNA-ChIP experiment to detect the association of MARVELD1 with mRNA containing PTC.

GFP-tagged PTC-mRNA was immunoprecipitated with MARVELD1, UPF1 and SMG1 antibodies in 16HBE/siCtrl cells, whereas the PTC-mRNA did not immunoprecipitate with IgG (negative control). Interestingly, siRNA-mediated depletion of MARVELD1 in 16HBE cells led to an obvious reduction of PTC-mRNA in all the immunoprecipitates (Figure 5b). Statistical analysis showed significant difference in the amount of PTC-mRNA immunoprecipitated with MARVELD1, UPF1 and SMG1 antibodies between control and MARVELD1 knockdown groups (Figure 5d).

The observation was also confirmed in lung cancer cell line A549 expressing low level of MARVELD1. Shown as Figure 5c and 5e, MARVELD1 overexpression robustly increased the detected PTC-mRNA that was immunoprecipitated in A549/MARVELD1 cells. These results indicated that MARVELD1 associated with PTC-mRNA, and the association between PTC-mRNA and UPF1/SMG1 was enhanced by MARVELD1. Taken together, these findings suggested that epigenetic inactivation of *MARVELD1* gene will weaken NMD pathway in lung cancer, which may be required for lung tumorigenesis.

## Discussion

In this study, we reported the correlation between MARVELD1 silencing and lung cancer. Immunohistochemical analysis and Western blotting experiments showed that the down-regulation of MARVELD1 was associated with malignant progression of lung cancer (Figure 1, Table S2). Epigenetic silencing of *MARVELD1* gene was observed in low MARVELD1-expressing lung cancer cell lines (Figure 2). Therefore, we deducted that MARVELD1 silencing may be required for tumorigenesis and has a potential to be developed as a biomarker for malignant phenotype of lung cancer, which possesses considerable value in prognosis prediction, progression monitoring and treatment evaluating. For future application, it is critical to design reasonable clinical trial for evaluation of this biomarker. Not only Overall survival (OS) and Disease-free survival (DFS) should be evaluated in randomized, controlled trials, some key factors with regard to lung cancer, such as smoking history information, will be considered.

Hypermethylation of tumor suppressor genes and demethylation of oncogenes contribute to tumorigenesis. Cancer-linked hypermethylation and hypomethylation of gene promoter is often associated with cell proliferation^23^,^24^,^25^. MARVELD1 is a potential tumor suppressor, which negatively regulates proliferation of cancer cells^19^. In this study, we observed that MARVELD1 is down-regulated in lung cancer tissues, especially small-cell lung cancer tissues. In lung cancer cell lines, the methylation states of the CpGs in *MARVELD1* promoter region were found to be hypermethylated. Moreover, *MARVELD1* gene was also re-expressed following treatment with 5-aza-CdR, a potent demethylating agent, suggesting that status of DNA demethylation was important for the active expression of MARVELD1. In addition, we also found histone acetylation and DNA demethylation synergistically activated *MARVELD1* gene in lung cancer cells.

Previous results showed that MARVELD1 could suppress cell spreading and actin organization through regulation of pre-mRNA processing, a process parallel to NMD^20^. Moreover, MARVELD1 associated with potential NMD factor Importin β1, suggesting the possible function of MARVELD1 in NMD pathway^21^,^22^. Recent study in our lab further showed that MARVELD1 regulated NMD via modulating phosphorylation of UPF1^26^. Considering the aberrant regulation of MA*RVELD1* gene in lung cancer, therefore, we discussed the gene silencing mechanism of MARVELD1 and the relationship between epigenetically masked MARVELD1 and NMD function in lung cancer cells.

**Figure 6:**
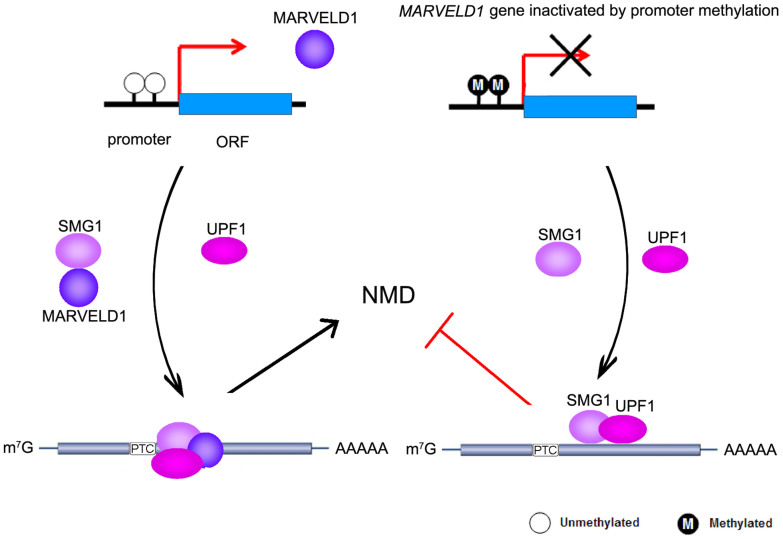
Working model. MARVELD1 promotes NMD pathway through enhancing the association between UPF1/SMG1 and PTC-containing mRNA in normal cells, while MARVELD1 silencing by epigenetic modification attenuates NMD in lung cancer cells.

Although NMD pathway has been extensively studied, the regulatory mechanism of NMD in cancer is still not well understood. In this study, we showed MARVELD1 co-localized and interacted with SMG1, the core kinase of the NMD machinery, in lung cancer cells. Moreover, we constructed the NMD reporter plasmid expressing PTC-containing truncated LRP1B-GFP mRNA (LRP1B exons 1–9 from lung cancer cell line QG56), and demonstrated that MARVELD1 bound PTC-mRNA as efficient as NMD core factor UPF1 and SMG1 by RNA-ChIP based reporter system. In addition, we also found MARVELD1 could enhance the association between NMD complex UPF1/SMG1 and PTC-containing mRNA, which suggested that MARVELD1 was involved in modulating the efficiency of NMD through interaction with SMG1. In summary, our study suggested the following working model (Figure 6). The reduced MARVELD1 expression, due to promoter hypermethylation, attenuated NMD, which indicated aberrant mRNA surveillance mechanism in lung cancer.

## Methods

### Cell culture and transfection

All cell lines used in this study were originally purchased from the American Type Culture Collection (ATCC, Manassas, VA, USA). The cells were cultured in RPM1640 medium supplemented with 10% fetal calf serum and 2 mM L-glutamine. Cells were transiently transfected with indicated siRNA or plasmids using Lipofectamine 2000 according to manufacturer's instructions (Invitrogen, Carlsbad, CA, USA). Sequences of siRNA used in this study were listed in Table S1.

### Plasmid constructions

To construct the GFP-tagged NMD reporter plasmid, truncated and full-length MARVELD1 promoter luciferase reporter plasmids, DNA fragments were amplified using primers in the Table S1. Then the DNA fragments were ligated into pEGFP-N1 and pGL3 Basic vector, respectively. The expression plasmids for MARVELD1 were constructed previously by our laboratory^18^.

### Quantitative real-time PCR

Quantification of human MARVELD1 mRNA was performed in a 20 μl mixture consisted of 10 μl 2× SYBR Green Mix, 0.2 μl RT product, 1 μl primer pair mix at a concentration of 5 pmol/ml for each primer and 8.8 μl sterile water. As internal control, GAPDH mRNA was performed in same condition except the primer. The experiment was set on ABI 7500 Real-Time PCR System (Foster City, CA, USA), PCR program was according to protocol's recommendation (10 minutes at 95°C for 1 cycle, 10 seconds at 95°C, 34 seconds at 60°C and 30 seconds at 72°C for 40 cycles, 10 minutes at 72°C for 1 cycle). Primers for qPCR are described in Table S1.

### Immunohistochemical analysis and and immunofluorescence assay

The immunohistochemical staining was performed as described previously^19^. 55 small cell lung carcinoma tissue samples were from small cell lung carcinoma tissue array LC802 (US Biomax, Rockville, MD) and lung carcinoma tissue array OD-CT-RsLug01-007 (Outdo Biotech, Shanghai, China). 139 paired lung cancer tissues were from human lung cancer array OD-CT-DgLug01-007, HLug-Ade150Sur-01 and HLug-Squ150Sur-01 (Outdo Biotech, Shanghai, China). Immunofluorescence assay was performed as described previously^20^.

### Bisulfite DNA sequencing

Bisulfite DNA sequencing was conducted as described previously^19^. Briefly, bisulfite modification of DNA was performed by following the protocol of Applied Biosystems methylSEQr Bisulfite Conversion kit. Bisulfite-treated DNA was amplified using nest PCR with primers described in Table S1. Amplified bisulfite-treated DNA was sequenced and compared with original sequence of *MARVELD1* gene.

### Luciferase assay

Luciferase assay was performed as described previously^27^. Cells were transfected with truncated *MARVELD1* promoter luciferase reporter plamids using Lipofectamine 2000 (Invitrogen, Carlsbad, CA, USA). Luciferase assays were performed by using the luciferase assay system (Promega, Madison, WI, USA); β-Galactosidase activity was used as an internal control. Luciferase activity was measured by using CytoFluorplate 4000 Luminescence Microplate Reader (ABI, Foster City, CA, USA).

### Co-IP assay and western blotting

Total 1 × 10^7^ cells were lysed in a fresh RIPA lysis buffer with protease inhibitors. The supernatants were collected and incubated with antibodies raised against MARVELD1 (Abcam, Cambridge, MA) at 4°C for 12 h. Protein A Sepharose CL-4B beads (GE, Piscataway, NJ, USA) were incubated with the mixture at 4°C for 2 h. Then the beads were washed 3 times with RIPA buffer, and the bound proteins were eluted with SDS-PAGE loading buffer. Western blotting was conducted as described previously^18^.

### RNA-ChIP assay

RNA ChIP assays were performed as described with minor modifications^28^. Briefly, cells were cross-linked by addition of formaldehyde to a final concentration of 1% (v/v). Glycine was added to 125 mM to quench cross-linking. The cells were resuspended in 500 μl Buffer A (5 mM PIPES, pH 8.0, 85 mM KCl, 0.5% *NP40,* protease inhibitors cocktail, 50 U/ml SUPERase). Raw nuclei were resuspended in 500 μl Buffer B (1% SDS, 10 mM EDTA, 50 mM Tris-HCl pH 8.1, protease inhibitors cocktail, 50 U/ml SUPERase), and sonicated for IP reaction with specific antibody. Each immune complex was washed five times in washing buffer and eluted by addition of 300 μl Elution buffer (10 mM Tris, pH 8.0, 5 mM EDTA, 1% SDS, 50 U/ml SUPERase). RNA was isolated with Trizol reagent and resuspended in 20 μl DEPC-treated water followed by DNaseI treatment. Further analysis was performed by using RT-PCR method.

## Author Contributions

M.S., S.W. and Y.L. (Yu Li) designed the study and drafted the manuscript. M.S., Y.Y. and J.S. performed the qRT-PCR, bisulfite DNA sequencing, Co-IP and RNA-ChIP assay. Y.Y., L.Y. and J.C. carried out luciferase assay, immunofluorescence and immunohistochemistry assay. Y.L. (Yiqun Li) carried out bioinformatics analysis. Y.L. (Yiqun Li), H.Z., F.H., Z.W. and N.H. helped to collect the data. All authors read and approved the final manuscript.

## Supplementary Material

Supplementary Information Figure S1
