# Supplementary material for: Biological and clinical significance of epigenetic silencing of MARVELD1 gene in lung cancer
Source: Sci Rep. 2014 Dec 18;4:7545. doi: 10.1038/srep07545 (PMC4269892; doi:10.1038/srep07545)
Supplement: Supplementary Information — Figure S1 [file srep07545-s1.pdf]

Supporting information

Biological and clinical significance of epigenetic silencing of MARVELD1 gene in lung cancer

Ming Shi[1], Shan Wang[1], Yuanfei Yao[1], Yi Qun Li[1], Hao Zhang[1], Fang Han[1], Huan Nie[1], Jie Su[1], Zeyu Wang[1], Lei Yue[1], Jingyan Cao[2] and Yu Li[1]*

[1]School of Life Science and Technology, Harbin Institute of Technology, Harbin, China

[2]Department of Medical Oncology, Harbin Medical University Cancer Hospital, Harbin, China

(a)

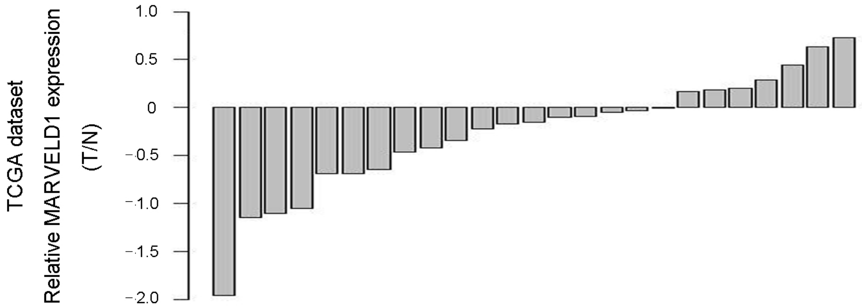

(b)

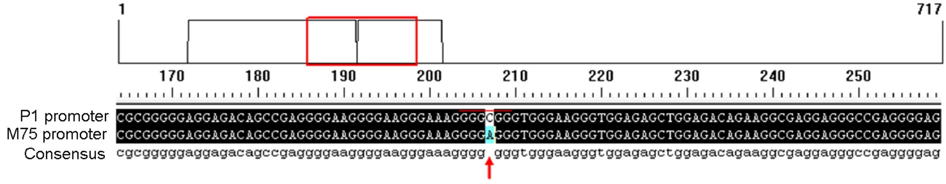

Figure S1 Analysis of MARVELD1 expression in NSCLC tissues and sequence alignment of MARVELD1 promoter

(a): analysis of MARVELD1 mRNA expression in NSCLC tissues and their matched adjacent lung tissues in TCGA dataset. n=25. (b): sequence alignment between wildtype P1 promoter and M75 promoter with a site-directed mutation

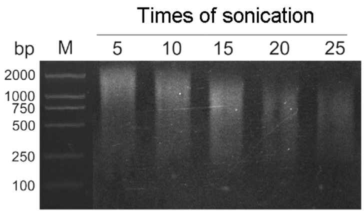

Figure S2 DNA fragmentation was generated from genome DNA by sonication.

Conditions for shearing of chromatin by sonication: 250 W, working 20 sec, resting 40 sec.

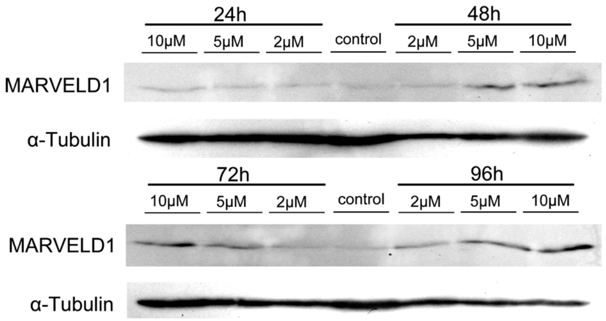

Figure S3 Effect of 5-aza-CdR on expression of MARVELD1 gene in A549.

Western blotting analysis showed the effect of 5-aza-CdR on MARVELD1 expression in A549 cells.

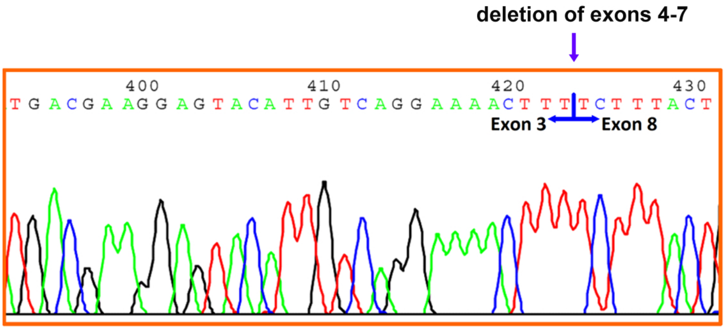

Figure S4 Part of Sequencing chromatogram of *LRP1B* gene Exons 1-9 from lung cancer cell line QG56.

A PTC was derived from deletion of exons 4-7 that was indicated by blue arrows.

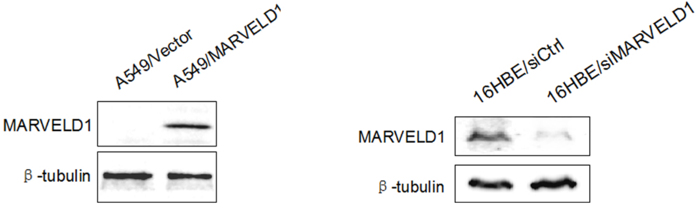

Figure S5 MARVELD1 expression was detected by western blotting analysis.

(a): A549 was transfected with MARVELD1 expressing plasmid and vector control. The overexpressed MARVELD1 was analyzed by western blotting. (b): 16HBE was transfected with siMARVELD1 and siCtrl. The reduced MARVELD1 was analyzed by western blotting.

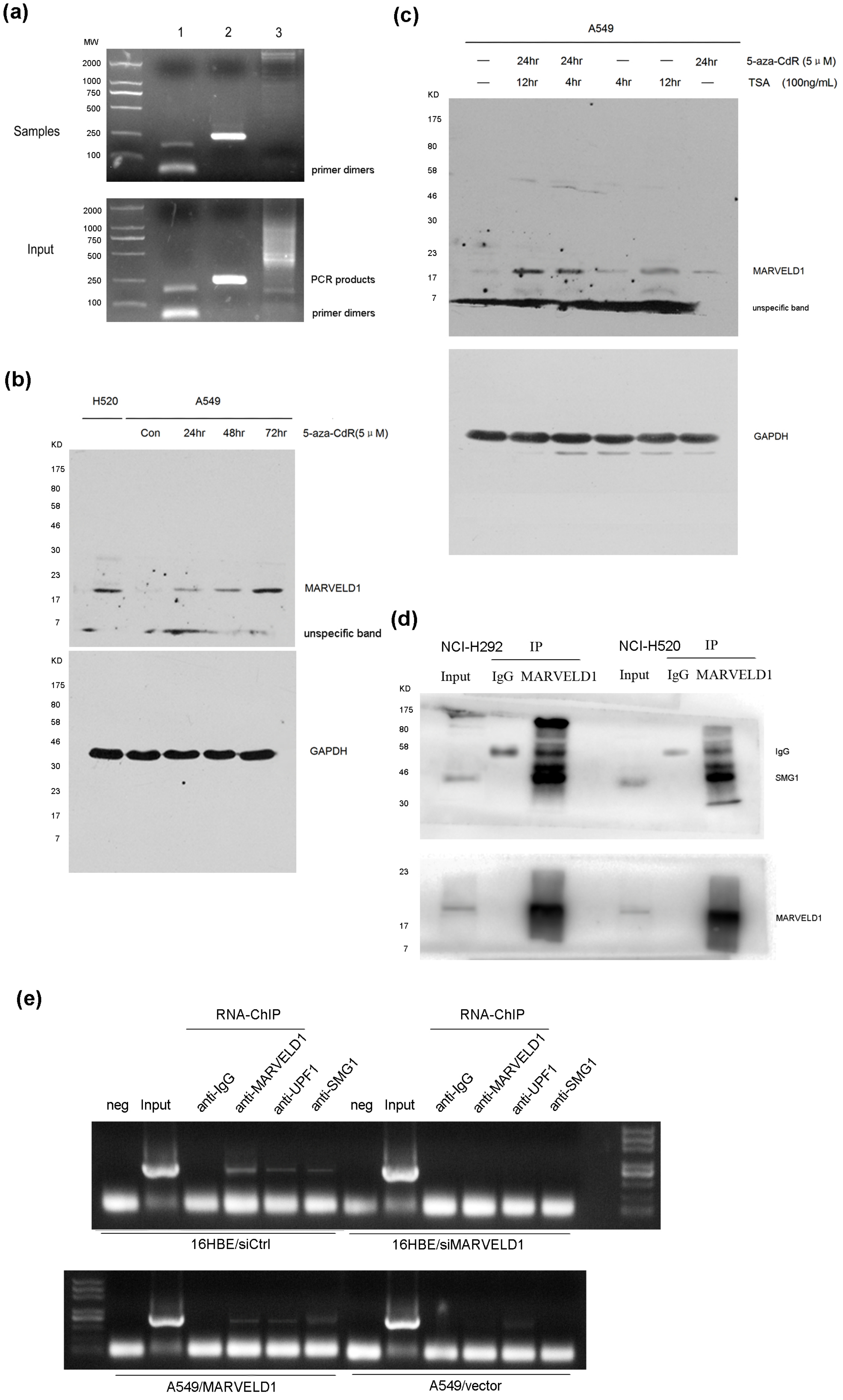

Figure S6 Full-length gels and blots

(a): Chromatin immunoprecipitation (IP) assay using H520 cell lysates. Chromatin was immunoprecipitated using antibody against Sp1 (refer to Figure 2d). (b,c): Western blotting analysis showed the effect of 5-aza-CdR (b) and the effect of 5-aza-CdR and TSA (c) on MARVELD1 expression in A549 cells (refer to Figure 3a, 3c). (d): Co-IP analysis showed the interaction between MARVELD1 and SMG1 in NCI-H292 and NCI-H520 cells. MARVELD1 and SMG1 were immunoprecipitated using antibody against MARVELD1. Anti-IgG was used as a negative control (refer to Figure 4b, 4c).(e): The association of GFP-tagged PTC-mRNA and proteins MARVELD1, UPF1 or SMG1 in 16HBE and A549 cells was evaluated in RNA-ChIP assay by using MARVELD1, UPF1 or SMG1 specific antibody. H_2O (neg) acted as a negative control in PCR assay (refer to Figure 5b, 5c).

Table S1 Oligo sequences

Quantitative RT-PCR	
MARVELD1	F: 5′ -GAG GAA GTA GAT TGC TGC CTC TAG-3′
	R: 5′ -GAG AGC GGT CGG TGT GAC CAG CTC TG-3′
GAPDH	F: 5′ - AAC AGC CTC AAG ATC ATC AGC-3′
	R: 5′ - GGA TGA TGT TCT GGA GAG CC-3′
Plasmid constructs	
MARVELD1/P1	F: 5′ -CGACGCGTAGGACGAAGGAATCCGAGCTG-3′
MARVELD1/P5	F: 5′ -CGACGCGTGGGCGCTATGGCAGGAAAG-3′
	R:5′ - GAAGATCTCCCACCCTTCCTCTCCTCTCTC-3′
Bisulfite DNA sequencing	
BS1	F: 5′ -GTG TTT TTG GTG GGT GAT G-3′
	R: 5′ -TCT TCC TCT CCT CCT TTC-3′
BS2	F: 5′ -GAT GTT TAG GTG ATA GTG G-3′
	R: 5′ -TAC TCC CCT CTT AAT CCT C-3′
siRNA	
siCtrl	F: 5′ -UUCUCCGAACGUGUCACGUTT-3′
	R: 5′ -ACGUGACACGUUCGGAGAATT-3′
siMARVELD1	F: 5′ -AUUGGAACCAGGCUUCUGGTT-3′
	R: 5′ -CCAGAAGCCUGGUUCCAAUTT-3′

Table S2. Correlation of MARVELD1 expression with clinicopathological features of lung carcinoma patients

Variable	All patients	Low	High	p value
Gender				0.428
Male	141	113	28	
Female	53	39	14	
Age (years)				0.541
≤60	85	63	22	
>60	109	86	23	
TNM grade				<0.001*
I	33	21	12	
II-III	64	62	2	

*P<0.001 was considered statistically extremely significant.

Low (- to +), High (+/++ to +++).
